# Rupture of the thoracic aorta associated with experimental *Angiostrongylus vasorum* infection in a dog

**DOI:** 10.1051/parasite/2012192189

**Published:** 2012-05-15

**Authors:** L.R. Mozzer, W.S. Lima

**Affiliations:** 1 Department of Parasitology, Federal University of Minas Gerais Av. Presidente Antônio Carlos 6627 31270-901 Belo Horizonte, MG Brazil

**Keywords:** *Angiostrongylus vasorum*, dog, thoracic aorta, hemothorax, *Angiostrongylus vasorum*, chien, aorte thoracique, hémothorax

## Abstract

This note describes the sudden death of a dog by the rupture of the thoracic aorta caused by the presence of *Angiostrongylus vasorum*. A female mongrel canine with a history of weight loss and exhaustion died two hours after clinical examination. At necropsy, performed one hour after death, showed the presence of clotted blood in the thoracic cavity. Haemothorax was diagnosed. The thoracic aorta wall was thin, congested and an abnormal hole in the wall was detected approximately 0.5 cm from the entrance to the diaphragm. From clotted blood collected from the thoracic cavity, 224 first stage larvae (L1) and 15 adults of *Angiostrongylus vasorum* were recovered alive. Also, from a blood clot found in the aorta, four adult females and 47 L1 larvae were recovered alive. Possibly, this parasite was responsible for the aortic rupture and death of the animal.

## Abstract (fr)

Cette note décrit la mort subite d’un chien par rupture de l’aorte thoracique causée par la présence d’*Angiostrongylus vasorum*. Un chien de race, avec une histoire de perte de poids et d’épuisement, est mort deux heures après l’examen clinique. La nécropsie, réalisée une heure après la mort, a montré la présence de sang coagulé dans la cavité thoracique. Un hémothorax a été diagnostiqué. La paroi de l’aorte thoracique était mince, congestionnée, et un orifice d’environ 0,5 cm a été détecté. Il se situait près de l’entrée de l’artère dans le diaphragme. 224 larves (L1) et 15 adultes d’*Angiostrongylus vasorum* ont été retrouvés vivants dans le sang coagulé recueilli dans la cavité thoracique. Par ailleurs, dans le sang coagulé contenu dans l’aorte thoracique ont été trouvées quatre femelles adultes et 47 larves (L1). Ce parasite est probablement responsable de la rupture de l’aorte et de la mort de l’animal.

*Angiostrongylus vasorum* (French Heartworm) is a nematode parasite of the right ventricle and pulmonary artery and its branches in wild and domestic canids (Verzberger-Epshtein *et al.*, 2008). It has been reported in Europe, North America, South America and Africa, covering tropical, subtropical and temperate region (Koch & Willesen, 2009). The life cycle of *A. vasorum* is heteroxenic, with terrestrial and aquatic snails as intermediate hosts. These mollusks are infected by first stage larvae (L1) by ingestion or penetration into the tegument. The infection of the definitive host occurs by ingestion of third stage larvae (L3). The main clinical signs are related to the cardiorespiratory system as dyspnea, tachycardia, arrhythmias, persistent dry cough and other clinical signs such as pyodermas, alopecia, weight loss, onychogryphosis, thrombosis, disseminated intravascular coagulation and death of the animal (Cury *et al.*, 2002; Morgan *et al.*, 2005). Although there have been studies involving the life cycle of the parasite, there are still mechanisms that are not well understood concerning the biology of *A. vasorum*. One of these mechanisms is erratic migration, which is sporadically mentioned in the literature (Perry *et al.*, 1991; Cury & Lima, 1996; Oliveira-Júnior *et al.*, 2004; Sasanelli *et al.*, 2008).

## Method

A female mongrel canine, 14 kg was inoculated by oral route with 2,800 infective larvae of *A. vasorum* to maintain the strain in the laboratory. Parasites were obtained from the tissue of an intermediate host *Omalonyx matheroni* maintained in laboratory and experimentally contaminated with the first-stage larvae (L1) of this nematode, following methodology Mozzer *et al.* (2011). The dog was born in the breeding facilities of the Federal University of Minas Gerais, Brazil under the management systems on animal well-being and according to the ethics committee of the university (CETEA/UFMG). The dog was subjected to physical examinations by a veterinarian before inoculation, and weekly from ten days postinoculation (dpi). Blood samples were taken before inoculation and twice a month from the day of inoculation. Fecal samples were taken daily from 25 dpi.

## Results

The first meeting of larvae in the feces of the dog was 35 dpi. At 39 dpi the animal walked with its back arched and was indifferent to its environment. The dog presented with a temperature of 38.1 °C, heart rate of 160 beats/minute, respiratory rate of 42 breaths/minute and pale pink, dry mucous membranes with a prolonged capillary refill time greater than two seconds. Clinical examination revealed loud bronchovesicular sounds on thoracic auscultation and attenuated cardiac sounds. Showed increased superficial cervical lymph nodes, chest sensitivity and pain from manipulation in this region.

Following the examination, blood samples were collected for laboratory tests, which showed the following values: 5,000,000 erythrocytes/mL, 11.70 g/dL hemoglobin, 38.05 % hematocrit, 158,000 platelet/mL and 26,000 leukocytes/mL. The differential leukocyte count was as follows: 6 % band neutrophils, 34 % segmented neutrophils, 22 % eosinophils, 35 % lymphocytes and 2 % monocytes. The animal had 90 mg/dL of fibrinogen. The prothrombin time was 11 seconds, and the partial activated thromboplastin time was 13 seconds.

The animal was died two hours after the clinical examination. From the necropsy performed one hour after death, it was observed that the mucosa and carcass were very pale. After retracting the skin, internal intercostal muscles showing suffusion points were found. Opening the thorax showed the presence of clotted blood in the thoracic cavity. When examining the circulatory system, approximately 15 cm of the thoracic aortic wall was irregular, thickened and firm. An abnormal hole in the wall of the thoracic aorta was detected approximately 0.5 cm from the entrance to the diaphragm. At this location, the aorta wall was thin and congested. From 980 ml of clotted blood collected from the chest cavity, 224 first stage (L1) larvae, 12 adult females and three adult males nematodes were recovered alive. From a clot found inside the aorta, 47 L1 larvae and four adult females were recovered alive. The parasitic nematodes were identified as *Angiostrongylus vasorum* (Lima *et al.*, 1985).

The heart was hypertrophied. The lungs had multiple nodules of different sizes scattered diffusely throughout the parenchyma. The diaphragm had suffusion points. The superficial cervical, auxiliary and mediastinum lymph nodes were enlarged and congested. Examination of the locomotion system showed no change, with bone strength within normal limits. A total of 118 adult males and 103 adult females of *A. vasorum* were observed and recovered.

## Discussion

The clinical, hematological and pathological changes found in the examined animal are consistent with canine angiostrongylosis and corroborate descriptions made in previous studies (Prestwood *et al.*, 1981, Perry *et al.*, 1991; Patteson *et al.*, 1993; Cury *et al.*, 2002, Schnyder *et al.*, 2010). The circulatory problems in infected animals are due to mechanical irritation in the arterial endothelium caused by the parasites. The eggs, larvae and adults cause inflammatory and thromboembolic pulmonary processes. Erratic migrations of larvae and adults to the kidneys, brain, eyes, femoral artery and bladder have been observed (Perry *et al.*, 1991; Cury & Lima, 1996; Oliveira-Júnior *et al.*, 2004; Denk *et al.*, 2009). Sudden death in dogs with angiostrongylosis may be caused by obstruction of the pulmonary artery or other major arteries or heart failure (Patteson *et al.*, 1993; Ramsey *et al.*, 1996; Chapman *et al.*, 2004; Garosi *et al.*, 2005; Staebler *et al.*, 2005; Wessmann *et al.*, 2006).

Sasanelli *et al.* (2008) described one case report in which hemothorax was caused by *A. vasorum* infection; however, this result does not explain the presence of L1 larvae in the pleural effusion seen in this study. Therefore, this is the first report of a fatal thoracic aortic rupture by hemothorax caused by adult and larval *A. vasorum* and accompanied by thrombus formation, local inflammation and weakness of the artery wall. The recovery of *A. vasorum* adults and larvae in organs other than those involved in the natural parasite life cycle has been described (Oliveira- Júnior *et al.*, 2004; Cury & Lima, 1995, 1996). The discovery of another ectopic location of the parasite is important for understanding *A. vasorum* pathogenesis because erratic migrations can cause death or injury in other tissues by changing their functionality and causing symptoms that increasingly prevent clinical diagnoses of angiostrongylosis. Moreover, it is clear that further studies focusing on disease pathogenesis and the actual life cycle within the definitive host are needed to clarify such migrations of the parasite.

The nonspecificity of clinical signs hinder the clinical diagnosis of the disease since it can be mistaken with other diseases leading to the misdiagnosis and an underestimation of the real parasite. The presence of males and females erratic parasites as well as the presence of the first larval stage within the aortic artery and the fluid found in the thoracic cavity showed the association with hemothorax and describes for the first time the rupture of the aortic artery. Continued bleeding following elective surgery in infected dogs has been reported in practice (Helm *et al.*, 2010) and these cases might be presented as emergencies. It is notable that the severity of bleeding correlates poorly with the extent of detectable coagulation abnormalities, such that some dogs exhibit severe signs with apparently normal coagulation profiles. Thus, screening for infection and performing a coagulation profile before surgery in known hyperendemic areas is indicated. It is fair to assume that increasing populations of dogs are at risk from *A. vasorum* infection. This note has relevance to warn doctors of small animals veterinary clinic’s that angiostrongylosis should be related within differential diagnoses in dogs with bleeding and cardiopulmonary disorders especially in enzootic areas.
